# Supplementary material for: A Transcription Factor Regulates Gene Expression in Chloroplasts
Source: Int J Mol Sci. 2021 Jun 24;22(13):6769. doi: 10.3390/ijms22136769 (PMC8268430; doi:10.3390/ijms22136769)
Supplement: Supplementary file 1 [file ijms-22-06769-s001.zip › ijms-1273677-supplementary.pdf]

SUPPORTING INFORMATION

```
                           *        20         *        40         *        60         *        80
AT5G63790.1 : ----------MDFALFSSISIFEINHKDPPIRRFIKTQNRILSTRKQQGTFPKMKAELNLPAGFRFHPTDEELVKFYLCR :  70
AT5G63790.2 : MNLPFVYKSSMDFALFSSISIFEINHKDPPIRRFIKTQNRILSTRKQQGTFPKMKAELNLPAGFRFHPTDEELVKFYLCR :  80
                        MDFALFSSISIFEINHKDPPIRRFIKTQNRILSTRKQQGTFPKMKAELNLPAGFRFHPTDEELVKFYLCR

                           *       100         *       120         *       140         *       160
AT5G63790.1 : RCASEPINVPVIAEIDLYKFNPWELPEMALYGEKEWYFFSHRDRKYPNGSRPNRAAGTGYWKATGADKPIGKPKTLGIKK : 150
AT5G63790.2 : RCASEPINVPVIAEIDLYKFNPWELPEMALYGEKEWYFFSHRDRKYPNGSRPNRAAGTGYWKATGADKPIGKPKTLGIKK : 160
              RCASEPINVPVIAEIDLYKFNPWELPEMALYGEKEWYFFSHRDRKYPNGSRPNRAAGTGYWKATGADKPIGKPKTLGIKK

                           *       180         *       200         *       220         *       240
AT5G63790.1 : ALVFYAGKAPKGIKTNWIMHEYRLANVDRSASTNKKNNLRLDDWVLCRIYNKKGTMEKYLPAAAEKPTEKMSTSDSRCSS : 230
AT5G63790.2 : ALVFYAGKAPKGIKTNWIMHEYRLANVDRSASTNKKNNLRLDDWVLCRIYNKKGTMEKYLPAAAEKPTEKMSTSDSRCSS : 240
              ALVFYAGKAPKGIKTNWIMHEYRLANVDRSASTNKKNNLRLDDWVLCRIYNKKGTMEKYLPAAAEKPTEKMSTSDSRCSS

                           *       260         *       280         *       300         *       320
AT5G63790.1 : HVISPDVTCSDNWEVESEPKWINLEDALEAFNDDTSMFSSIGLLQNDAFVPQFQYQSSDFVDSFQDPFEQKPFLNWNFAP : 310
AT5G63790.2 : HVISPDVTCSDNWEVESEPKWINLEDALEAFNDDTSMFSSIGLLQNDAFVPQFQYQSSDFVDSFQDPFEQKPFLNWNFAP : 320
              HVISPDVTCSDNWEVESEPKWINLEDALEAFNDDTSMFSSIGLLQNDAFVPQFQYQSSDFVDSFQDPFEQKPFLNWNFAP

AT5G63790.1 : QG : 312
AT5G63790.2 : QG : 322
              QG
```

Figure S1. Alignment of two NAC102 isoforms

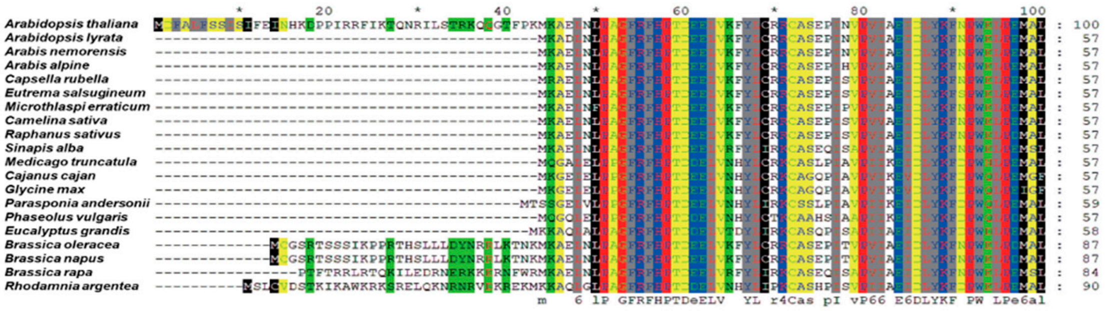

Figure S2. Alignment of NAC102 orthologs

The N-Terminal sequences of NAC102 orthologs from *Arabidopsis thaliana* (NP_201184.2), *Arabidopsis lyrata* (XP_002866576.1), *Arabis nemorensis* (VVB17923.1), *Arabis alpine* (KFK28073.1), *Capsella rubella* (XP_006280952.1), *Eutrema salsugineum* (XP_006394242.1), *Microthlaspi erraticum* (CAA7034898.1), *Camelina sativa* (XP_010460978.1), *Raphanus sativus* (XP_018439406.1), *Sinapis alba* (KAF8081687.1), *Medicago truncatula* (RHN43155.1), *Cajanus cajan* (KYP61874.1), *Glycine max* (NP_001341065.1), *Parasponia andersonii* (PON61827.1), *Phaseolus vulgaris* (XP_007159873.1), *Eucalyptus grandis* (KCW54084.1), *Brassica oleracea* (VDD27371.1), *Brassica napus* (CDY39846.1), *Brassica rapa* (VDC78532.1), and *Rhodamnia argentea* (XP_030551358.1) were aligned using ClustalW.

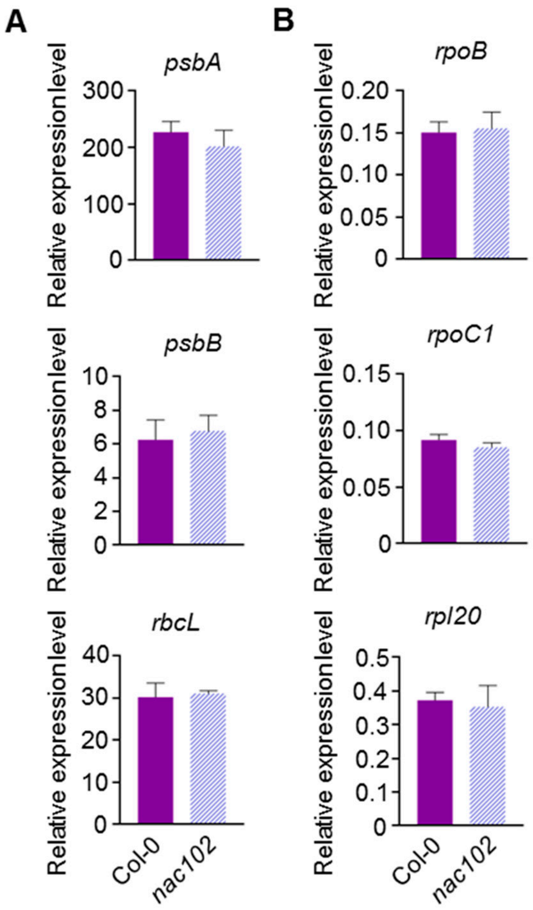

Figure S3. The chloroplast gene expression in the *nac102* mutant

The relative expression levels of the chloroplast genes were determined by qRT-PCR analysis using *EF1α* as the normalizer. (A) The PEP-dependent genes. (B) The NEP-dependent genes. Data represent mean ± SD (n = 3). The statistical significances were determined using Student's t-test (*$p < 0.05$, **$p < 0.01$, and ***$p < 0.001$).

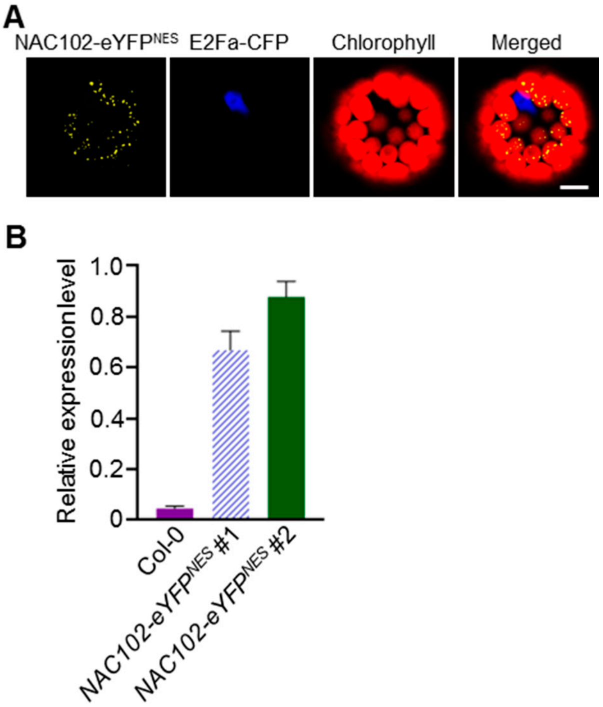

Figure S4. Construction of *NAC102-eYFP^{NES}* overexpression lines

(A) Subcellular localization of the NAC102-eYFP^{NES}. The fusion proteins were transiently expressed in *Arabidopsis* protoplasts. E2Fa-CFP was used as the nucleus marker and the chlorophyll fluorescence was used as the chloroplast marker. The pictures were captured using confocal microscopy. Bar = 10 μm. (B) The relative expression levels of *NAC102* in Col-0 and the *NAC102-eYFP^{NES}* transgenic lines were determined by qPCR using *EF1α* as the normalizer. The data are represented as means ± SD (n = 3).

Table S1. List of primers used in this study

Primer name	Sequence (5'-3')	Applications
NAC102-eYFP F1	CAATTACATTTACAATTACCATGGACTTTGCTCTCTTCTC	Fusion proteins
NAC102-eYFP F2	CAATTACATTTACAATTACCATGAACCTCCCCTTCGTTTA	
NAC102-eYFP R1	TGGGCCGCCGCCGGAGCCGCCGCCTGGCCCTTGAGGAGCAAAATTCC	
eYFP F1	CCAGGCGGCGGCGGCTCCGGCGGCGGCCCAGTGAGCAAGGGCGAGGAGCT	Fusion proteins
eYFP R	CCGGGTCTTAATTAACTCTCTAGATTACTTGTACAGCTCGTCCA	
NAC102-eYFP^{NES} R1	TTAGTCTAAAGTGAGGCGCTCAAGTGGAGGGAGACACTTGTACAGCTCGTCCATGC	Fusion proteins
NAC102-eYFP^{NES} R2	CCGGGTCTTAATTAACTCTCTAGATTAGTCTAAAGTGAGGCGCT	
NAC102$^{\triangle N43}$-eYFP F	CAATTACATTTACAATTACCATGAAGGCGGAGTTGAATTT	Fusion proteins
NAC102^{N43}-eYFP R	AGCTCCTCGCCCTTGCTCACTTTTGGAAAAGTACCTTGTT	Fusion proteins
NAC102^{N60}-eYFP R	AGCTCCTCGCCCTTGCTCACGTCCGTCGGATGAAATCGGA	Fusion proteins
NAC102^{N80}-eYFP R	AGCTCCTCGCCCTTGCTCACCGGAACGTTAATCGGTTCTG	Fusion proteins
eYFP F2	GTGAGCAAGGGCGAGGAGCT	Fusion proteins
EF1α-qPCR F	AAATACTCCAAGGCTAGGTACG	qPCR
EF1α-qPCR R	AAATGGGATTTTGTCAGGGTTG	
NAC102-qPCR F	GAGATGTGCGTCAGAACCGA	qPCR
NAC102-qPCR R	TTTCTCACCGTACAACGCCA	
psbA-qPCR F	TGAGCACAACATTCTTATGCAC	qPCR
psbA-qPCR R	AGTTACCAAGGAACCATGCATA	
psbB-qPCR F	GTTTTGGCGCATTTCATGTAAC	qPCR
psbB-qPCR R	TGGAATAGGCCCGCTAATATAC	
rbcL-qPCR F	TCTGGTGGAGATCATATTCACG	qPCR
rbcL-qPCR R	ATCATCTCCAAAGATCTCGGTC	
rpoB-qPCR F	GCGAATCGAGCTTTAATGAGTT	qPCR
rpoB-qPCR R	AAACGATCTTCTCAGTGTCAGT	
rpoC1-qPCR F	GTGCTATCCGAGAACAATTAGC	qPCR
rpoC1-qPCR R	GACATAAAACCATCCATTCGGG	
rpl20-qPCR F	TATTTGCATCAAGCTTTCGAGG	qPCR
rpl20-qPCR R	TGATCCATAAACGGCGAAAATC	
NAC102 BD F	CTCAGAGGAGGACCTGCATATGGACTTTGCTCTCTTCTC	Y2H
NAC102 BD R	CTAGTTATGCGGCCGCTGCAGCCCTTGAGGA	

	GCAAAATTCC	
rpoA AD F	ACGTACCAGATTACGCTCATATGGTTCGAGAG AAAGTCAA	Y2H
rpoA AD R	CAGTATCTACGATTCATCTGCAGTTTTTTTTCT AGAATGTCTA	
rpoB AD F	ACGTACCAGATTACGCTCATATGCTTGGGGAT GAAAAAGA	Y2H
rpoB AD R	CAGTATCTACGATTCATCTGCAGAACTTCCTT CCTATTAATCT	
rpoC1 AD F	ACGTACCAGATTACGCTCATATGATCGATCGG TATAAACA	Y2H
rpoC1 AD R	CAGTATCTACGATTCATCTGCAGGGTATCATAT GAACAGGCTT	
rpoC2 AD F	ACGTACCAGATTACGCTCATATGGCGGAACG GGCCAATCT	Y2H
rpoC2 AD R	CAGTATCTACGATTCATCTGCAGAATCCTAGA AAAGTCAGATT	
rpoTp AD F	ACGTACCAGATTACGCTCATATGGCTTCCGCT GCGGCGGC	Y2H
rpoTp AD R	CAGTATCTACGATTCATCTGCAGGTTGAAGAA GTACTGTGATT	
rpoTmp AD F	ACGTACCAGATTACGCTCATATGTCCAGTGCT CAAACCCC	Y2H
rpoTmp AD R	CAGTATCTACGATTCATCTGCAGGTTGAAGAA ATAAGGTGAAT	
